# Optimization of Fat-Reduced Puff Pastry Using Response Surface Methodology

**DOI:** 10.3390/foods6020015

**Published:** 2017-02-22

**Authors:** Christoph Silow, Emanuele Zannini, Claudia Axel, Markus C. E. Belz, Elke K. Arendt

**Affiliations:** School of Food and Nutritional Sciences, University College Cork, Cork T12 YN60, Ireland; christoph.silow@gmx.de (C.S.); E.Zannini@ucc.ie (E.Z.); c.axel@umail.ucc.ie (C.A.); m.belz@umail.ucc.ie (M.C.E.B.)

**Keywords:** laminated bakery product, wheat, lipids, texture analyzer, sensory

## Abstract

Puff pastry is a high-fat bakery product with fat playing a key role, both during the production process and in the final pastry. In this study, response surface methodology (RSM) was successfully used to evaluate puff pastry quality for the development of a fat-reduced version. The technological parameters modified included the level of roll-in fat, the number of fat layers (50–200) and the final thickness (1.0–3.5 mm) of the laminated dough. Quality characteristics of puff pastry were measured using the Texture Analyzer with an attached Extended Craft Knife (ECK) and Multiple Puncture Probe (MPP), the VolScan and the C-Cell imaging system. The number of fat layers and final dough thickness, in combination with the amount of roll-in fat, had a significant impact on the internal and external structural quality parameters. With technological changes alone, a fat-reduced (≥30%) puff pastry was developed. The qualities of fat-reduced puff pastries were comparable to conventional full-fat (33 wt %) products. A sensory acceptance test revealed no significant differences in taste of fatness or ‘liking of mouthfeel’. Additionally, the fat-reduced puff pastry resulted in a significant (*p* < 0.05) positive correlation to ‘liking of flavor’ and overall acceptance by the assessors.

## 1. Introduction

The history of layered doughs is thousands of years old and even puff pastry in its present form has been known for several hundred years [1]. Puff pastry is a light and flaky pastry made of laminated dough which can be topped or filled, sweet or savory, enabling a large range of product variations. Unlike other laminated baked goods such as croissants and Danish pastry, puff pastry is made of unleavened dough without any other rising agents [2].

According to the so-called French method—the most common way to produce puff pastry—a piece of fat (traditionally butter) is wrapped with basic dough, which is then folded and sheeted several times to obtain a multi-layered dough. To date, no global uniform standard exists for the preparation of puff pastry or related products. For instance, in the German literature, the French method describes that a piece of dough is wrapped in fat. The Dutch or Scottish method describes a rapid preparation of puff pastry with lower volume and quality [2]. The cold fat is cut into cubes which are added and mixed with the basic dough before the lamination, which results in discontinuous layers. However, with combinations of different, repeated folding steps, products with numerous layers, usually from 48 to 256, can be obtained. Typically, these multi-layered doughs have a relatively high amount of fat (20%–35% on total mass).

Primarily, high costs and the difficult handling of the traditionally used butter during industrial processing initiated the development of specifically manufactured fat blends [3]. These fat blends are derived mainly from vegetable oils and fats, offering a better process ability (fat plasticity). Thus, nowadays, they are preferred for commercial puff pastry production. Nevertheless, artisanal bakeries still prefer butter.

The functional roles of fat in puff pastry making are to separate the many thin dough layers from each other and, after melting, to protect the starch granules from gelatinization [4]. During the baking process, the water located in the dough vaporizes and generates steam which expands but cannot pass the coagulated gluten network within the dough layers [4]. This expansion of steam between the dough layers causes the rise of the puff pastry. In addition, fat is essential as a flavor carrier and gives the final product its specific characteristics, such as a good structure, texture and mouth feel [5,6].

To conclude, fat plays a key role in puff pastry production and cannot be replaced or reduced entirely without adversely affecting its production and the product quality [7]. This is a challenge for the puff pastry producing industry which is forced to reduce its fat and calorie content due to the increasing global trend towards the production of more wholesome foods. Thus, the food industry bears great responsibility to produce healthy foods and therefore, cost-effective strategies are needed for the successful reduction of fat in their products. In principle, two strategies exist to accomplish fat reduction in puff pastry: either by reducing the total amount of roll-in fat used or by replacing the fat component with a low-fat or fat-free substance.

Previous studies in which fat was substituted by another solid substance having a fat content have been described for Danish pastries. In this case, the roll-in fat was completely replaced by frozen dough or shaved ice [7]. Other studies focused on the reduction of saturated and trans-fatty acids in the roll-in fat [8,9]. Further studies dealt with the use of maltodextrins, pentosans or other hydrocolloids to produce fat-reduced margarine-like emulsions containing aqueous gels [10,11].

These so-called fat replacers such as hydrocolloids belong to the group of food additives which must appear in the list of ingredients according to the EU regulation No. 1169/2011 [12]. However, increasingly, the application of food additives does not fit in the current health-conscious consumer life-style. Moreover, fat reduction in foods has become increasingly popular in recent decades. Multiple reports have demonstrated that the consumption of low-fat products can lower the energy and fat intake and may help with long-term weight control and the maintenance of good general health.

The aim of the present study was to reduce the total fat content of puff pastry as much as possible without adversely affecting the product quality by only reducing the amount of roll-in fat and adjusting the processing parameters. Hence, only technological parameters were modified, guaranteeing a food additive-free labelling. Changes included the number of fat layers and the final thickness of the laminated dough. Confocal laser scanning microscopy was used to investigate the microstructure of the unbaked laminated dough. Finally, response surface methodology (RSM) was used to determine the optimal conditions processing for fat-reduced puff pastry with quality characteristics comparable to full-fat standard products. This strategy describes an alternative approach for fat-reduction on a cost-neutral basis, making it also accessible for implementation in the bakery industry. In addition, sensory evaluation of fat-reduced puff pastry compared to full-fat puff pastry products was performed with a sensory acceptance test, which provided insights into which attributes are important when describing puff pastry quality. 

## 2. Experimental

### 2.1. Materials

The ingredients used in this study were wheat flour (Grand Moulins de Paris, France, type T45, moisture 13.5%, protein 11.5%), salt (Glacia British Salt Limited, UK), commercially available lemon juice (Tesco) and tap water. The vegetable fat blend 8324 G. Olmatech Cobalt (s.a. Aigremont n.v., Awirs-Flémalle, Belgium) had a composition of 66% palm stearin and 34% rapeseed oil and was used as roll-in fat (RIF) for puff pastry preparation. The solid fat content (SFC) of the fat blend was provided by the retailer (Temperature (°C)/SFC (%): 10.0/48.2; 20.0/36.0; 30.0/25.1; 40.0/15.5).

### 2.2. Puff Pastry Production

Puff pastries were produced according to the so-called French method, wherein a piece of fat is wrapped with basic dough and then folded several times to obtain a multi-layered dough. The basic recipe was taken from different German bakery literature [13,14] and slightly modified. Instead of a butter/flour mix, a vegetable fat blend (66% palm stearin, 34% rapeseed oil) was used as roll-in fat (RIF).

The dough was prepared in a standard mixer (Model: A200, Hobart Mfg. Co. Ltd., London, UK) with a kneading hook. The formulation for the basic dough consisted of 1000 g flour, 21 g salt, 15 g lemon juice and 510 g cooled tap water (12.5 °C–16.0 °C). Flour and salt were premixed. Subsequently, the liquids were added and the mixing was carried out at a first speed (48 rpm) for 2 min and for a further 3 min at a second speed (90 rpm). Dough temperature after mixing was in the range of about 22.5 ± 1.5 °C. A portion of 1500 g basic dough was wrapped in a plastic bag to prevent dehydration and left to rest at room temperature for 20 min. If not clearly identifiable by the context, hereafter, the term ‘basic dough’ refers to the mixture of ingredients (flour, salt, lemon juice and water) before incorporating the RIF; ‘laminated dough’ refers to the laminated mix of basic dough and RIF before baking; and ‘pastry’ refers to the final baked product.

Calculations of the required RIF (in grams, g) were based on the fat percentage of the laminated dough (*FC_LD_ = (RIF)/(m_BD_ + RIF)* × 100) before baking by rearranging this equation accordingly:

(1) $$RIF\left(g\right)=\frac{\left(m_{BD}\times FC_{LD}\right)}{\left(100-FC_{LC}\right)}$$

where *m_BD_* is the mass of basic dough in g and *FC_LD_* is the fat content in laminated dough in percent (*w/w*).

RIF, according to Table 1, was formed to a square and sheeted on a Rondo sheeter (Model: SSO 605, Seewer AG, Burgdorf, Switzerland).

The basic dough was also formed to a square and sheeted to a size just twice as large as the corresponding fat block. The fat block was placed on the dough and encased with this dough while the edges were sealed together. There are two conventional ways of folding laminated dough to produce puff pastry: a single turn (three theoretical fat + four dough layers) and a double turn (four theoretical fat + five dough layers). With variable combinations of single and double turns, different numbers of theoretical layers can be obtained: e.g., one double turn followed by one single turn which results in 12 theoretical fat layers (4 × 3) or, if these steps are repeated another time, 144 theoretical fat layers (12 × 12) will be created (Table 2).

Once the fat was enclosed by the dough, it was laminated to a thickness of 10 mm. The number of turns to obtain the appropriate number of fat layers and resting periods are shown in Table 2. Before each turn, the laminated dough was turned 90° in horizontal orientation and subsequently rolled down to 10 mm.

Layered dough was rolled down gradually to the desired final thickness (1.00–3.50 mm) at which stage two passages were performed. After a rest of 20 min, samples of 10 × 10 cm were cut out randomly and were allowed to rest at 4 °C overnight within baking paper in an airtight bag. The puff pastry control had a full-fat content (0% fat reduction), 144 theoretical fat layers and a final thickness of 2.50 mm.

After refrigeration, overnight samples were allowed to reach room temperature and baked afterwards on trays with baking paper at 210 °C in a top- and bottom-heated, unventilated, preheated deck oven (Model: MIWE condo INT 01/01, Michael Wenz GmbH, Arnstein, Germany). Optimal baking times had been determined based on the degree of browning by an experienced baker in preliminary trials. Baking times varied for the different final thicknesses: (baking time (min)/final thickness (mm): 7.0/1.00; 9.5/1.50; 11.0/1.75; 12.5/2.00; 14.0/2.25; 15.0/2.50; 15.5/3.00; 16.0/3.50).

### 2.3. Physicochemical Analysis of Puff Pastry

After baking and cooling to room temperature (~30 min), physicochemical analysis of puff pastry was conducted. Therefore, samples were weighed, and specific volume and maximum lift (maximum height) were evaluated using a VolScan Profiler (Stable Micro Systems, Godalming, UK). Turning speed of the VolScan Profiler was set to one round per second; vertical step of the laser was set to 1 mm. Product length and width were determined manually over the sample middle using an electronic caliper. 

Texture analyses of puff pastry were carried out 2 h after baking. The firmness of puff pastry was determined by penetration tests using a Texture Analyzer TA-XTplus (Stable Micro Systems Ltd., Godalming, UK). Two different methods were set up using an Extended Craft Knife (ECK) as well as a Multiple Puncture Probe (MPP; both Stable Micro Systems Ltd., Godalming, UK) equipped with a load cell of 30 kg (Force Sensitivity: 1 g). Firmness was obtained from the ‘work of shear’, which represents the area under the characteristic compression curve. The tests were conducted under the following conditions: test mode: compression, test speed: 5.00 mm·s^−1^ (for ECK); 2.00 mm·s^−1^ (for MPP), post-test speed: 10.00 mm·s^−1^, target mode: distance, distance: 64.500 mm, trigger type: button, return distance: 65 mm, contact force: 15 g; software: Texture Exponent 32, version 4.0 (Stable Micro Systems Ltd., Godalming, UK).

For ECK measurements, the sample was cut breadthwise through its center. To standardize all ECK results, firmness values were divided by their respective width (in mm) and multiplied by 100. MPP was used with nine needles from which eight needles were arranged in the outer circle and one needle in the center of the probe. The needle arrangement was determined in preliminary trials (data not shown), since tests with all needles compressed the samples rather than penetrated them.

For internal structure characterization of baked puff pastry, an image analysis of the cross sections of puff pastry halves was conducted using a C-Cell imaging system (Calibre Control International Ltd., Warrington, UK). Images of the samples, cut with the ECK, were taken. The number of cells and slice brightness were the parameters to describe the internal structure. Furthermore, the ratios of number of cells/height and number of cells/slice area were calculated to include the relationship of the average (avg) height and the area of the puff pastry cross sections to the structure.

Following data evaluation, limits and ranges of the physicochemical parameters were set for the subsequent RSM optimization procedure.

### 2.4. Experimental Design

RSM was applied to the experimental data to evaluate the effect of the independent variables (fat reduction, number of fat layers and final dough thickness) on the dependent variables (firmness (ECK), firmness (MPP), specific volume, number of cells and slice brightness). Herein, optimum parameter levels could be determined.

A circumscribed, three-dimensional central composite design was developed featuring variations in fat reduction (ranging from 0% to 50%, based on fat percentage of the laminated dough), number of fat layers (ranging from 50 to 200) and final dough thickness (ranging from 1.00 mm to 3.50 mm). The upper and lower limits of these levels were selected based on general empirical values from the literature and on preliminary trials conducted (data not shown). A total of 17 trials were carried out, comprising eight for the factorial, six for the axial and three as central points.

In addition, the response data of a further 37 randomized trials, within the above-mentioned ranges, were included in the design. The response of each of the investigated parameters was analyzed by fitting cubic models to the data with least square regression in order to identify the significant effects of the variations in parameter levels on the responses (*p* < 0.05). To visualize the overall trend, three-dimensional graphs for the models were used.

### 2.5. Sensory Evaluation of Puff Pastry

The sensory acceptance test was performed according to Stone, Bleibaum, and Thomas [15] and Stone and Sidel [16]. The sensory panel consisted of 60 untrained assessors. After tasting, they had to fill in a questionnaire in which they were asked for personal details and for questions regarding the puff pastry products. The test was conducted for puff pastry control, improved puff pastry control and fat-reduced puff pastry. All samples were prepared as described above. After cooling, the samples were cut in two equal halves, labelled with a random 3-digit code and served to the assessors at the same time on a white plate in a randomized order. The experiment was conducted at room temperature in panel booths which conformed to International Standards [17]. All samples were presented in duplicate with a different sample order [18]. The assessors were asked to assess, on a continuous line scale from 1 to 10 cm, the following attributes: ‘liking of appearance’, ‘liking of color’, ‘liking of volume’, ‘liking of flavor’, ‘liking of mouthfeel/texture’ and overall acceptability (hedonic). The assessors then participated in a ranking descriptive analysis (RDA) [19] using the consensus list of sensory descriptors including the number of layers, fatness, saltiness, moisture, firmness, crispiness and off-flavor (intensity), which was also measured on a 10 cm line scale. The sensory acceptance test was done in duplicate [18].

All data were evaluated with ANOVA (analysis of variance)-Partial least squares regression (APLSR) using Unscrambler software version 9.7 (CAMO Software AS, Oslo, Norway).

### 2.6. Confocal Laser Scanning Microscope

For confocal laser scanning microscopy (CLSM) (Biorad, Herts, UK), sample preparation was conducted with frozen unbaked laminated dough. Briefly, samples were stored at −18 °C and thin slices of approximately 0.2 to 0.5 mm of the dough cross section were cut off using a scalpel. The slices were stained with 0.2% aqueous Rhodamin B solution (Sigma-Aldrich, Dublin, Ireland) on a glass plate for 2 min and rinsed with water afterwards. Samples were examined with a FV300 confocal laser-scanning system mounted on an Olympus IX80 inverted microscope with a 10× dry objective (Olympus, Center Valley, PA, USA), using *λ*_ex_ = 543 nm.

### 2.7. Compositional Analysis

Compositional analysis was performed for the improved puff pastry control (0% fat reduction, 81 fat layers, 2.50 mm final thickness) and the fat-reduced puff pastry (40% fat reduction, 48 fat layers, 2.25 mm final thickness). Five random samples per recipe were frozen in a plastic bag to −32 °C for 30 min using a Blast Freezer (BF 35, Foster Cross Refrigeration Ltd., Norfolk, UK). Instantly, frozen samples were blended using a Büchi Mixer B-400 (BÜCHI Labortechnik AG, Flawil, Switzerland) to ascertain homogeneity. Crude fat was determined by extraction of a 6.5 g homogenized sample using a Soxtec Manual Extraction Unit 2055 (Foss, Hillerød, Denmark). For determining the ash content, approximately 8.0 g of blended sample was heated to 600 °C for 12 h [20]. Protein content was determined from approximately 1.0 g of the homogenized sample using the Kjeldahl method [21]. Moisture content was determined according to the approved AACC method 44–15.02. [22]. Total carbohydrates were calculated by difference. All determinations were done in triplicate.

### 2.8. Statistical Analysis

Design Expert Version 7 (Stat-Ease Inc., Minneapolis, MN, USA) was used for experimental design and to generate surface response plots that permitted evaluation of the linear, quadratic and interactive effects of independent variables on the selected dependent variables and to optimize puff pastry formulations for levels of 0% and 40% fat reduction.

All values were tested for outliers according to Grubbs [23]. For comparison reasons, Statistica 7.1 (StatSoft, Tulsa, OK, USA) was used to carry out statistical analysis on the test results. A normality test (Shapiro–Wilk) was followed by an all pair wise multiple comparison procedure (Fisher LSD, post hoc test) to evaluate significant differences.

## 3. Results and Discussion

### 3.1. Baking Trials and Experimental Design

In the present study, RSM was used to evaluate the effect of the technological parameters which were, number of fat layers, final dough thickness and, especially, the level of roll-in fat on puff pastry quality. Therefore, 17 trials of the central composite design with variations in fat reduction, number of fat layers and final dough thickness were carried out. The evaluation of the response data showed that these 17 trials did not provide sufficient information to describe the correlations of the independent variables adequately. Hence, the response data of a further 37 randomized trials within the design ranges were included in the design and data evaluation was repeated.

In Table 3, the ANOVA results for quality parameters of puff pastry are presented.

The best fit model, which evaluated the effect of the independent variables on the response, was chosen. Thus, the significance of the lack of fit error term, coefficient *R*^2^, coefficient of variation (CV) and model significance were used to judge the adequacy of the model fit. The predictive models developed for firmness (ECK), firmness (MPP), specific volume, number of cells and slice brightness of puff pastry were considered adequate since they had a non-significant lack of fit and had satisfactory levels of *R*^2^, CV and model significance (Table 3).

**Figure 1:**
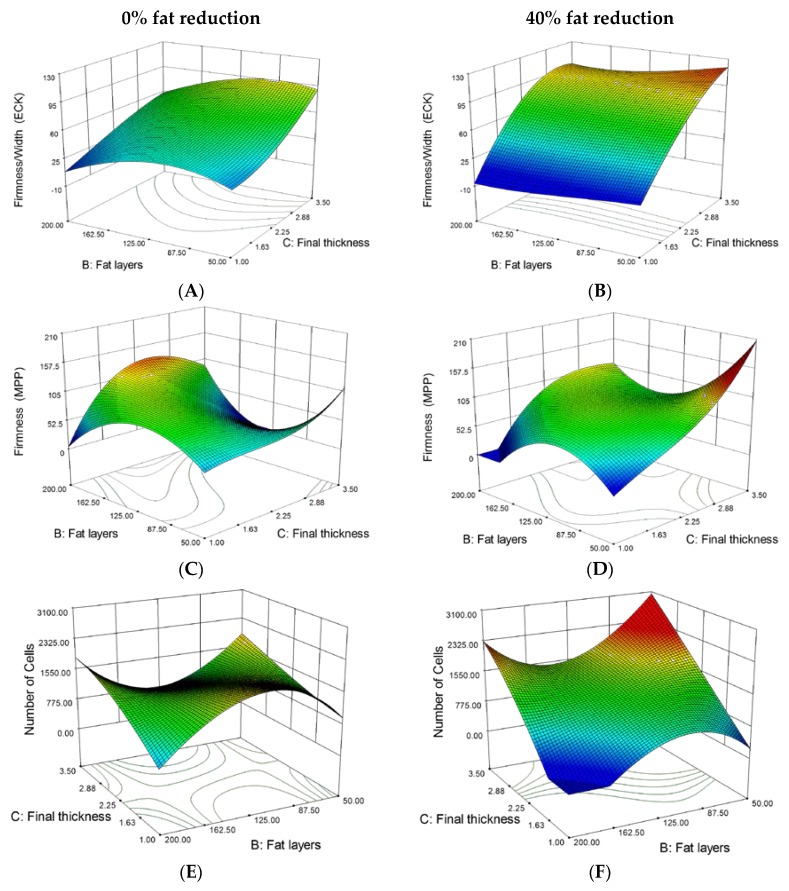

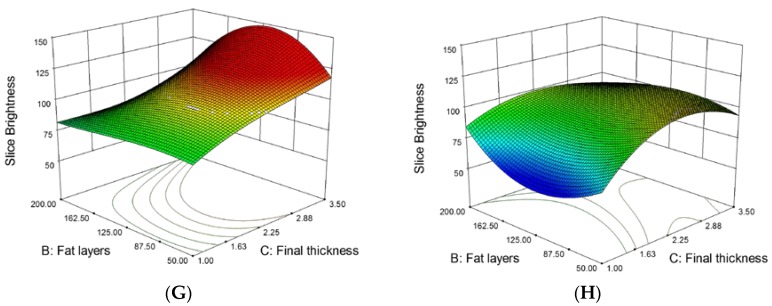
Response surface plots: Effect of final dough thickness and number of fat layers on firmness (Extended Craft Knife) (**A**,**B**), firmness (Multiple Puncture Probe) (**C**,**D**), number of cells (**E**,**F**) and slice brightness (**G**,**H**) of puff pastry at levels of 0% and 40% fat reduction.

RSM was further used for the generation of response surface plots, which are a helpful tool to better understand the link between each factor and its response. Therefore, the effect of the two factors—the number of layers and final thickness—on one specific response were displayed in three-dimensional view while keeping fat reduction (third factor) as fixed values (0%, 40%). Eight selected surface plots are presented in Figure 1.

### 3.2. Firmness

**Figure 2:**
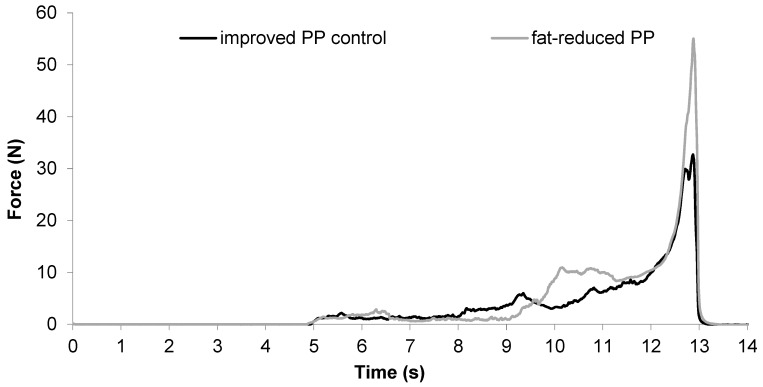
Force–time plot of 40% fat-reduced puff pastry (48 layers, 2.25 mm) and improved puff pastry control (81 layers, 2.50 mm).

In order to satisfy consumers’ preferences, puff pastry must have an acceptable firmness, internal structure and texture. Firmness is one of the main parameters describing the internal structure of the baked puff pastry. The method using a thin sharp blade (ECK)—imitates best the initial bite into a puff pastry. In contrast, the MPP hits the surface of the sample just at the very beginning and at the very end of the penetration which measures internal structure. Firmness is defined as the work required to cut (ECK) and to penetrate (MPP) the whole puff pastry sample. Typical force–time curves displaying firmness measurements (ECK) are shown in Figure 2.

As shown in Figure 1A,B, an increasing final thickness and decreasing number of layers led to firmer puff pastry. This effect is stronger for fat-reduced puff pastries. Firmness of the puff pastry varied significantly among the experiments, ranging from 7.0 to 124.5 N×s/mm for ECK measurements and 18.7 to 144.5 N×s for MPP (Table 4).

Comparison of the analytical values (range of responses, Table 4) and the subsequent sensorial evaluation by an experienced baker gave a range of firmness (ECK) values between 50 and 110 Ns/mm, relating to an acceptable firmness and texture of the puff pastry samples. Firmness values out of this range were related to puff pastries with poor internal structure and mouthfeel. The thinner the final paste was sheeted and the more dough layers it contained, the thinner were the single dough layers in the end. It seems that many thin dough layers led to a lower firmness (ECK) in the product than few thicker dough layers. Firmness (MPP) for low-fat contents (minus 40%) basically followed the same trend and increased with increasing final thickness and decreasing number of layers (Figure 1D).

For full-fat puff pastry, the highest firmness (MPP) was obtained for high numbers of fat layers and medium final thicknesses (Figure 1C). For a lower number of layers, the firmness of full-fat puff pastry decreased. This is probably due to the higher fat content. During the baking process, the melted fat is partially moving into the dough layers [3,4] and crystallizes again when cooling down after baking. The fat crystals in the final product cause a softening of the dough layers by lubrication and make them more tender [3,5]. This softening effect increases for higher fat contents [24]. The findings are in accordance with the results reported by Baardseth et al. [25] who investigated different types and concentrations (350, 500 and 650 g/kg dough) of shortenings in Danish pastry. Firmness measurements were conducted with a Kramer shear cell and compared to results of a trained sensory panel. It was found that firmness decreases when the shortening concentration increases. Both sensory analysis and textural measurements were in good agreement. Sternhagen and Hoseney [26] reported contrary results analyzing the firmness of croissants with different levels of roll-in fat (15%, 20% and 25% dough based) using a compression test and a 36 mm diameter plunger. They could not find any significant differences in firmness. Keeping in mind that croissants and Danish pastry are made with leavened dough and additional ingredients, these results are only partially comparable to those of puff pastry, but give a good indication.

### 3.3. Number of Cells/Slice Brightness

**Figure 3:**
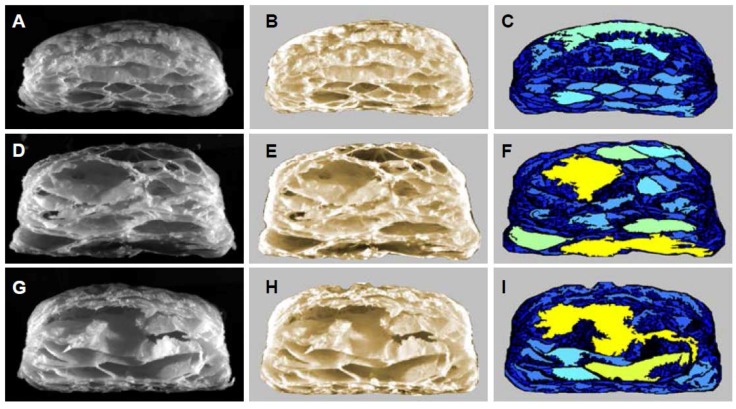
C-Cell images from cross sections of baked puff pastry with 40% fat reduction, 48 layers, 2.00 mm (first row), 0% fat reduction, 81 layers, 2.50 mm (second row) and 0% fat reduction 144 layers, 2.50 mm (third row), raw images (**A**,**D**,**G**), brightness image (**B**,**E**,**H**) and cell image (**C**,**F**,**I**).

In addition to firmness, the layered structure and exceptional flakiness of puff pastry are two of the most important characteristics influencing consumers’ choice [27]. Moreover, the internal structure of the baked puff pastry is directly related to the mouthfeel and crispiness [8]. Figure 3 shows raw images (A,D,G), taken with the C-Cell, which are representative of baked puff pastry samples with a well-developed internal structure.

Number of cells (Figure 3C,F,I) and slice brightness (Figure 3B,E,H) values were found to be the parameters which correlated best and allowed a prediction of the cellular and layered structure of the inner pastry.

In general, a larger number of cells is a good indication of a good internal puff pastry structure (data not shown). This is due to the fact that a higher number of cells represents a higher degree of crosslinking of the single dough layers, but also a larger cross-sectional area in the product. On the other hand, a higher number of cells within the same cross-sectional area implies that the cells are smaller and, thus, the internal structure is better. With an average of 1273 cells, the puff pastry control showed an acceptable internal structure (Table 4). The number of cells of 40% fat-reduced puff pastry at higher final thickness values has a minimum at about 140 layers and increases to both sides along the number of layers (Figure 1F). In theory, it should be assumed that more cells are created by higher numbers of dough and fat layers. However, in puff pastry with 100 or more dough layers, they were probably not separated properly by the fat layers to create cells which are big enough to be detected by the C-Cell system. The actually existing number of layers in puff pastry is well below the theoretically possible number, as observed by Noll et al. [28] and Telloke [29]. This effect can be seen in Figure 3 for full-fat puff pastries with 81 fat layers (second row) and 144 fat layers (third row).

Since each cell is encased by a cell wall of dough, more cells lead to a higher number of cell walls. The light reflected by these cell walls makes the product cross section appear brighter (Figure 3B,E,H). Thus, a high number of cells in the cross sectional area of the product basically results in high brightness values since the slice brightness positively correlates to the number and the thickness of dough cell walls. Unlike in bread analysis, the slice brightness value cannot be used as an independent parameter for puff pastry quality and has to be considered in conjunction with the other analytical parameters. The values might be false positive and the result is limited in information if, for example, products consist of only few thick dough layers. According to the RSM model (Figure 1G), the highest slice brightness values for full-fat puff pastry were obtained for high final thicknesses and about 140 layers. However, the slice brightness in relation to the number of layers for full-fat puff pastry seems to follow almost exactly the opposite trend to the number of cells as with increasing numbers of fat layers, lower numbers of cells were shown in the response surface plot (Figure 1E), which would result into lower values in brightness. The 40% fat-reduced puff pastry slice brightness achieved its highest values in the area of around 90 fat layers and a final thickness of 2.30 mm and decreased towards the sides (Figure 1H).

Conclusively, the number of cells and slice brightness values followed different trends depending on the amount of RIF used, which reflects the challenge in finding the best optimum.

### 3.4. Optimization of Process Variables

Optimum levels for the number of layers and final thickness were determined by superimposing the surface plots for all response variables using Design Expert software [30]. The regions that best satisfy all the quality requirements were selected as optimum conditions. For this reason, the responses of the physicochemical analysis were compared to the results and impressions of the sensorial analyses and thereof, the threshold values and ranges for qualitatively acceptable puff pastries were defined (Table 4). After evaluation of all data, minimum firmness (ECK and MPP), maximum number of cells and maximum slice brightness were the main quality criteria for the puff pastry optimization.

First, it was determined whether the puff pastry control may be improved by using the RSM. Therefore, fat reduction was set to 0%. An overview of all further limits and ranges for the optimization process of improved puff pastry control is given in Table 4. Lower and upper limits for the independent and dependent variables and the optimization target (‘maximize’, ‘minimize’ or ‘in range’) were set. These limitations resulted in the zone of the optimum conditions [31]. Taking into account all these limitations (Table 4), the optimization revealed few possible combinations for the parameters, number of fat layers and final thickness, for an improved puff pastry control (data not shown). One of these obtained combinations (82 layers, 2.48 mm thickness) was chosen for experimentally testing the optimized process. In practice, the technical capabilities to achieve the predicted number of fat layers and final thickness are limited by the method of preparation (folding steps) and the dough sheeter (roller gap). Thus, an improved puff pastry control with the closest feasible parameters, 81 layers and 2.50 mm final thickness, was prepared and analyzed. Afterwards, the results were compared to those predicted by the mathematical RSM model (Table 4). The measured values for the number of cells and the ratios, number of cells/height and number of cells/slice area, for the improved puff pastry control corresponded well to the predictions (RSM), while the predicted (RSM) value for firmness (MPP) was overestimated. Values for firmness (ECK), specific volume and slice brightness were lower than predicted by the Design Expert software. The RSM model does not include all possible external factors that might influence the final products and is therefore only an approximation. Hence, not all analyzed data can exactly match the values predicted by the mathematical RSM model.

However, to summarize, by using RSM modelling the improved puff pastry control (81 layers, 2.50 mm final thickness) gave better results compared to the puff pastry control (144 layers, 2.50 mm final thickness). In particular, firmness (ECK) decreased significantly and specific volume and number of cells increased significantly (*p* < 0.05) for the improved puff pastry control compared to the puff pastry control. This quality improvement of the puff pastry control by changing the number of layers was confirmed later by sensory evaluation.

After the successful optimization for the improved puff pastry control for the maximum fat reduction in puff pastry, a desired fat reduction of 40% was considered, while all further settings remained the same as before (Table 4). In this case, 46 layers and 2.27 mm thickness were obtained as one of the optimum operating points. Finally, to match the technical limits, fat-reduced puff pastry with 48 layers and 2.25 mm final thickness was baked and analyzed. The predicted and measured results are presented in Table 4. For 40% fat-reduced puff pastry, measurements of firmness (ECK), specific volume, number of cells and slice brightness corresponded well with the predictions of the RSM model, but slightly lower firmness (MPP) was measured than predicted.

### 3.5. Compositional Analysis

Compositional analysis was conducted for the optimized puff pastries: fat-reduced puff pastry and the improved puff pastry control (Table 5).

Fat content in the baked product was 29.0 ± 0.6 g/100 g and 45.0 ± 0.9 g/100 g for fat-reduced puff pastry and the improved puff pastry control, respectively. This is equivalent to a total fat reduction of 36%. Thus, the actual fat reduction in the fat-reduced puff pastry was slightly lower than calculated before (cf. Table 1) which was probably due the production process. The rough edges of the dough wide sides were trimmed before each further lamination. Finally, the total ratio shifts in favor of the fat since the removed edges contained mainly dough.

According to EU Regulation (EC) No. 1924/2006 on nutrition and health claims made on foods, a product can be stated as “reduced in fat” where the reduction in (fat) content is at least 30% compared to a similar product [33]. Since the total fat content in the fat-reduced puff pastry was reduced by more than 30%, this fat-reduced puff pastry can be claimed “reduced in fat”.

### 3.6. Confocal Laser Scanning Microscope

**Figure 4:**
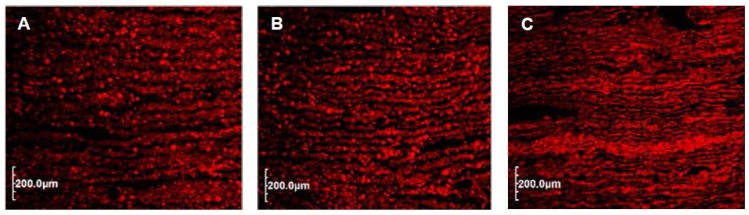
Confocal Laser Scanning Microscope representations of cross sections of laminated puff pastry dough (unbaked); protein network and starch granules (red) of the dough layers and intermediate fat layers (black). (**A**) 40% fat-reduced puff pastry (48 layers, 2.25 mm); (**B**) improved puff pastry control (81 layers, 2.50 mm); (**C**) puff pastry control (144 layers 2.50 mm).

To get a better understanding of the puff pastry dough, cross sections of the frozen samples were stained and examined with help of the CLSM. The micrographs (Figure 4) show the single dough and fat layers.

The dough layers (proteins, gluten network) appear red and the brighter red spots represent the starch granules. Generally, wheat starch can be separated into two fractions based on their granule size. Larger A-granules ranging from ~15 to 40 µm in diameter and smaller B-granules with a diameter range of ~1–10 µm [34]. Starch granules of both fractions are relatively uniformly distributed within the dough layers (Figure 4). Since the fat was not stained by the fluorescent dye, it appears as black. The parallel arrangement of the different layers and the variation in the thickness of the fat and dough layers can also be seen well. Since all micrographs have the same magnification, it is clearly recognizable that the number and thickness of the dough and fat layers vary.

Due to its well-developed gluten network, wheat dough is a viscoelastic material, even when containing several fat layers, and does not show ideal plastic behavior [35]. Hence, details on thickness do not represent the actual thickness of the laminated dough as the dough is expanding in the vertical direction (thickness) while contracting in the horizontal direction after passing the roller gap. For example, the actual thicknesses of the laminated doughs were 3.3 ± 0.1 mm for the 2.25 mm gap (final thickness) and 3.6 ± 0.1 mm for the 2.50 mm gap. For this reason, roller speed and number of passes were kept constant during all trials.

If the thickness of the dough is divided by the number of theoretical fat and dough layers, the theoretical thickness of a single layer can be obtained. A dough thickness of 3.3 mm divided by 97 layers (48 fat + 49 dough layers) gives an average thickness of 34 μm per layer. In this regard, for a final thickness of 2.50 mm (dough: 3.6 ± 0.1 mm), 81 and 144 fat layers, an average thickness per layer of 22 μm and 12 μm, respectively, will be obtained. According to Noll et al. [28], after the final lamination, every dough and fat layer in the puff pastry dough is thinner than a sheet of silk paper.

In the present study, the amount of fat used was reduced without replacing it with other ingredients. Hence, the volume of RIF which is available for building the fat layers was reduced and the thickness of fat layers decreased while the thickness of the dough layers increased, keeping the number of layers and final thickness constant. Assuming now that the dough layers expand more in the vertical direction and that they have a larger volume than the fat layers, the dough layers should be thicker than the fat layers. This is well visible in Figure 4A.

Apparently, there is a better distribution of the fat layers in the improved puff pastry control (Figure 4B) than for the 144 fat layers of the puff pastry control (Figure 4C). Here, the fat layers are too thin, broken at many points and probably cannot separate the dough layers from each other which ultimately led to poorer product quality [29].

### 3.7. Sensory Evaluation of Puff Pastry

A total of 60 panelists from 14 nations (28 Ireland, 9 Germany, 8 France, 15 other) participated in the sensory acceptance test. A total of 34 participants were female and the average age was 30 ± 11 years. Forty out of 60 panelists stated that they eat puff pastry monthly or even more often and two had never eaten it. Furthermore, 55% of the panelists had consumed fat-reduced food products previously.

**Figure 5:**
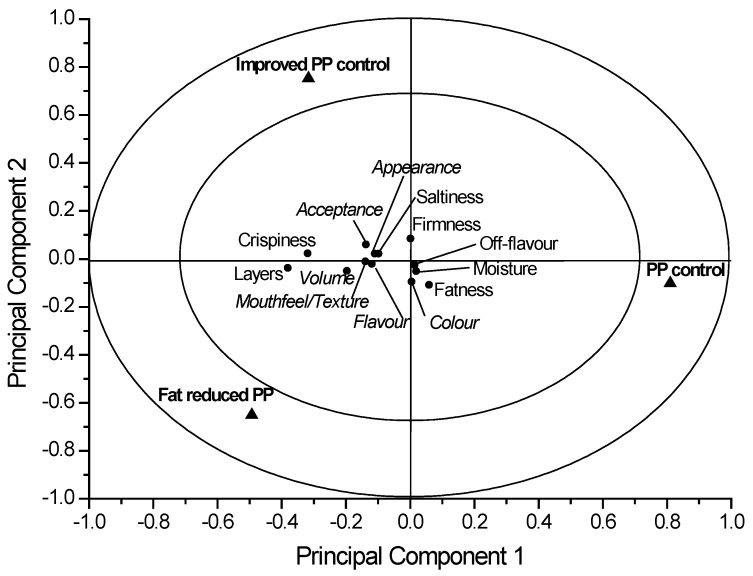
ANOVA-partial least squares regression (APLSR) correlation loadings plot for puff pastry (PP) samples. ▲ = samples, ● = sensory attributes (cursive style: hedonic attributes, not cursive: intensity attributes).

The results of the sensory evaluation are presented as an APLSR plot (Figure 5) in conjunction with the ANOVA values (Table 6).

In the right hand quadrant of the APLSR plot, the puff pastry control can be seen and in the opposite quadrant, the improved puff pastry control and fat-reduced puff pastry are located. The sensory attributes are accumulated in the plot center, whereby a sample puff pastry control shows opposite properties.

In detail, the puff pastry control was scored significantly (*p* < 0.05) lower in ‘liking of appearance’. Furthermore, positive trends were observed for the improved puff pastry control and the fat-reduced puff pastry in ‘liking of appearance’ (Table 6). Therefore, the attribute ‘liking of appearance’ was improved by the two new formulations.

Seemingly, no effect on color was found between the three puff pastry recipes since no significant assessment for ‘liking of color’ by the assessors was found.

Data for the puff pastry control also showed a very significant (*p* < 0.01) negative correlation to ‘liking of volume’, and for the improved puff pastry control and the fat-reduced puff pastry, a significant (*p* < 0.05) positive correlation was observed (Table 6). The analytical results (Table 4) show a significant higher volume for the two new puff pastry formulations which the panelists preferred. Contrary results were achieved in the study by Simovic et al. [8]. In this case, high fat puff pastry (55% margarine type MLT2) with a 45 min rest period gave a significantly increased volume and firmness which the panelists assessed as excellent quality. It is noteworthy that Simovic et al. [8] did not achieve their results by reducing the fat technologically as in the present study. However, in both studies, the panelists preferred puff pastries with higher volumes which showed that varying the number of fat layers and final thickness of puff pastry affects the sensory attribute ‘liking of the volume’.

Panelists assessed a significant negative correlation for puff pastry control (*p* < 0.05) to the ‘liking of mouthfeel/texture’ (Table 6).

Puff pastries are characterized by the laminated structure formed by layers (Figure 4) whereby the number of layers is an important sensory criterion. The puff pastry control contains, in theory, more layers than the improved puff pastry control and the fat-reduced puff pastry. However, the sensorial assessment of the puff pastry control showed a high significantly (*p* < 0.001) negative correlation to the number of layers (Table 6). The improved puff pastry control and the fat-reduced puff pastry were, however, significantly (*p* < 0.05, *p* < 0.01) positively correlated with the number of layers. The dough layers in the puff pastry control probably adhered to each other due to fat layers being too thin, as previously mentioned.

The attribute fatness, which represents the greasy mouthfeel of the puff pastry, was just associated with the sample puff pastry control and not to the improved puff pastry control which contains the same amount of fat. However, for all three samples, no significant differences in fatness were achieved (Table 6).

For the sensory attribute saltiness, no significant differences could be obtained which is consistent with the puff pastry formulations (cf. Table 1). The salt content in the basic dough was equal for full-fat and the fat-reduced puff pastry. However, due to the varied production processes, the absolute salt content in fat-reduced puff pastry was slightly higher since the dough and fat ratio shifts in favor of the dough. 

Inverse instrumental results for firmness were achieved, whereby the fat-reduced puff pastry had significantly higher values (*p* < 0.05) than the puff pastry control and the improved puff pastry control (Table 4).

The attribute crispiness is an important characteristic of puff pastry, which is mainly caused by the properties of the dough layers. All analyzed puff pastry samples could be positively correlated (*p* < 0.001, *p* < 0.05) to the attribute crispiness.

All samples were baked fresh on the sensory day. No significant positive correlations to off-flavor were determined by the assessors.

The main criteria associated with the repeated purchase of a food product are ‘liking of flavor’ and an overall satisfaction with the quality parameters. No correlation was found between the ‘liking of flavor’ and the overall acceptability for the puff pastry control (*p* < 0.05, *p* < 0.01). The modified formulations, the improved puff pastry control and fat-reduced puff pastry, obtained from the RSM modelling, resulted in a significant (*p* < 0.05) positive correlation between flavor and overall acceptability. Thus, the modified puff pastry formulations were clearly preferred in taste. The sensory acceptance test showed that RIF could be reduced by 36% without adversely affecting the products when compared to conventional products with a high fat content of 33 wt. % With the help of the RSM model and the Design Expert software, it was finally possible to reduce the fat content in puff pastry solely by changing the two technological parameters: the number of layers and final thickness.

## 4. Conclusions

Analytical methods for measuring the quality characteristics (firmness, specific volume, number of cells and slice brightness) of puff pastry were applied, including a Texture Analyzer attached with ECK and MPP, VolScan and a C-Cell image system. In particular, the parameters of the number of layers and final dough thickness were determined to play an important role as technological parameters which can be modified to allow a reduction in RIF. Using the RSM design as a tool for the optimization of fat-reduced puff pastry, with consideration of the independent parameters—fat reduction, number of fat layers and final thickness—was successful. The optimized parameters for fat-reduced puff pastry were 48 layers and 2.25 mm final thickness. This demonstrates that a reduction in the fat content in puff pastry is achievable with technological changes only and without the addition of fat replacers or fat-mimicking substances. As shown through sensory analysis, puff pastry products with a reduced fat content (36% reduced) can be produced with the best possible quality characteristics when compared to conventional products.

## Figures and Tables

**Table 1:** Used roll-in fat and fat content in laminated dough (LD).

Basic Dough (g)	Roll-in Fat (g)	Laminated Dough (LD) (g)	Fat Content in LD (g)	Fat Reduction (g)
1500	740	2240	33.0	0
1500	494	1994	24.8	25
1500	371	1871	19.8	40
1500	297	1797	16.5	50
1500	184	1684	10.9	67

**Table 2:** Theoretical number of fat layers and sequence of turns.

Fat Layers	Sequence of Turns *
12	4-RP30-3
36	4-RP30-3-RP90-3
48	4-RP30-4-RP90-3
64	4-RP30-4-RP90-4
81	3-RP30-3-RP90-3-RP30-3
108	3-RP30-3-RP90-3-RP30-4
128	4-RP30-4-RP90-4-RP30-2
144	4-RP30-3-RP90-4-RP30-3
192	4-RP30-4-RP90-4-RP30-3
256	4-RP30-4-RP90-4-RP30-4

* double turn (4), single turn (3), simple fold (2), resting period of 30 min (RP30) and 90 min (RP90).

**Table 3:** Analysis of variance (ANOVA) for evaluation of models for quality parameters of puff pastry. Coefficient of Variation: CV.

Response	Source	Sum of Squares	*F* Value	*p* Value
Firmness/Width (ECK)	Model	28,338.43	21.98	<0.0001
	Residual	2307.26		
*R*^2^ = 0.92	Lack of fit	1257.91	0.65	0.8130
CV (%) = 12.43	Pure error	1049.35		
Firmness (MPP)	Model	26,812.75	8.74	<0.0001
	Residual	3230.47		
*R*^2^ = 0.89	Lack of fit	2291.63	1.63	0.2492
CV (%) = 17.29	Pure error	938.83		
Specific Volume	Model	135.48	6.91	<0.0001
	Residual	34.05		
*R*^2^ = 0.80	Lack of fit	26.59	2.04	0.1026
CV (%) = 10.93	Pure error	7.46		
Number of Cells	Model	6.404 × 10^6^	10.07	<0.0001
	Residual	7.364 × 10^5^		
*R*^2^ = 0.89	Lack of fit	5.822 × 10^5^	2.16	0.1381
CV (%) = 13.81	Pure error	1.542 × 10^5^		
Slice Brightness	Model	8054.35	17.24	<0.0001
	Residual	540.99		
*R*^2^ = 0.94	Lack of fit	389.57	1.47	0.2977
CV (%) = 4.90	Pure error	151.42		

**Table 4:** Optimum ranges for independent parameters and predicted (by Response Surface Methodology) and measured values for the responses of puff pastry (PP) at 0% and 40% fat reduction.

	Settings for Optimization			Range of Experimental Design	PP Control	Improved PP Control		Fat-Reduced PP	
	Target for Optimization	Lower Limit	Upper Limit		Standard Dough	Predicted Parameters	Chosen Parameters	Predicted Parameters	Chosen Parameters
Fat reduction (%)	target = 0 *	0	40	0–50	0.0	0.0	0.0		
	target = 40 **	0	40	0–50				40.0	40.0
Fat layers	in range	36	144	12–256	144	82	81	46	48
Final thickness (mm)	in range	1.0	3.5	1.0–4.5	2.50	2.48	2.50	2.27	2.25
				**Range of Responses**	**Measured Values**	**Predicted Values**	**Measured Values**	**Predicted Values**	**Measured Values**
Firmness*100/Width (ECK) (N×s/mm)	minimize	50	110	7.0–124.5	65.2 ± 6.0 ^b^	63.9	54.6 ± 4.8 ^c^	80.2	80.4 ± 6.6 ^a^
Firmness (MPP) (N×s)	minimize	50	110	18.7–144.5	62.5 ± 4.8 ^b^	50.0	59.4 ± 8.1 ^b^	78.8	69.4 ± 5.9 ^a^
Specific Volume calc. (mL/g)	in range	7	13	5.3–13.3	6.6 ± 0.8 ^c^	8.8	8.3 ± 1.0 ^b^	10.3	10.4 ± 0.9 ^a^
Number of Cells	maximize	1200	2307	213–2307	1273 ± 105 ^c^	1431	1396 ± 264 ^b^	1672	1742 ± 245 ^a^
Slice Brightness	maximize	80.0	122.8	57.0–122.8	116.3 ± 8.5 ^a^	120.1	106.5 ± 7.8 ^b^	107.7	102.6 ± 11.1 ^b^
Number of Cells/Height (avg) (1/mm)	maximize	25.0	39.9	16.6–39.9	37.0 ± 4.2 ^a^	36.0	33.3 ± 3.5 ^b^	35.0	36.3 ± 4.2 ^a^
Number of Cells/Slice Area (1/mm^2^)	in range	0.450	0.600	0.382–0.606	0.519 ± 0.050 ^a^	0.515	0.484 ± 0.047 ^b^	0.508	0.508 ± 0.049 ^a^

Target for optimization * for improved puff pastry control; ** for fat reduced puff pastry, values are means ± SD; values in one row followed by the same lowercase letter are not significantly different (*p* < 0.05). Standard Deviation: SD.

**Table 5:** Compositional analysis of puff pastry (PP).

Composition	Improved PP Control	Fat-Reduced PP	Reference PP *
Protein (g/100 g)	5.4 ± 0.1	6.7 ± 0.1	6.7
Carbohydrates (g/100 g)	42.5 ± 0.7	52.6 ± 1.2	42.8
Fat (g/100 g)	45.0 ± 0.9	29.0 ± 0.6	33.2
Moisture (g/100 g)	6.0 ± 0.4	10.2 ± 1.3	13.4
Ash (g/100 g)	1.1 ± 0.1	1.5 ± 0.1	1.5

Values are means ± SD of three measurements. * Nutrient analysis survey of biscuits, buns, cakes and pastries—Food Standards Agency Analytical report [32].

**Table 6:** Significance of regression coefficients (ANOVA values) for the correlation of hedonic and intensity sensory terms for puff pastry (PP) formulations.

Sample	PP Control	Improved PP Control	Fat-Reduced PP
Liking of Appearance	−0.0495 *	0.1225 ns	0.0861 ns
Liking of Color	0.9384 ns	−0.9396 ns	−0.9376 ns
Liking of Volume	−0.0015 **	0.0415 *	0.0227 *
Liking of Flavor	−0.0195 *	0.1247 ns	0.0292 *
Liking of Mouthfeel/Texture	−0.0338 *	0.1132 ns	0.0641 ns
Overall Acceptability	−0.0103 **	0.1246 ns	0.0128 *
Layers	−1.0 × 10^−8^ ***	0.0223 *	0.0016 **
Fatness	0.4047 ns	−0.4934 ns	−0.3588 ns
Saltiness	−0.0994 ns	0.2083 ns	0.1073 ns
Moisture	0.7625 ns	−0.7719 ns	−0.7582 ns
Firmness	0.9872 ns	−0.9872 ns	−0.9872 ns
Crispiness	1.8 × 10^−7^ ***	0.0421 *	0.0007 ***
Off-flavor	0.8251 ns	−0.8295 ns	−0.8229 ns

Significance of regression coefficients; ns, not significant; * *p* < 0.050; ** *p* < 0.010; *** *p* < 0.0010.
